# A Modest Increase in ^11^C-PK11195-Positron Emission Tomography TSPO Binding in Depression Is Not Associated With Serum C-Reactive Protein or Body Mass Index

**DOI:** 10.1016/j.bpsc.2020.12.017

**Published:** 2021-07

**Authors:** Julia J. Schubert, Mattia Veronese, Tim D. Fryer, Roido Manavaki, Manfred G. Kitzbichler, Maria A. Nettis, Valeria Mondelli, Carmine M. Pariante, Edward T. Bullmore, NIMA Consortium, Dominika Wlazly, Amber Dickinson, Andy Foster, Clare Knight, Claire Leckey, Paul Morgan, Angharad Morgan, Caroline O'Hagan, Samuel Touchard, Shahid Khan, Phil Murphy, Christine Parker, Jai Patel, Jill Richardson, Paul Acton, Nigel Austin, Anindya Bhattacharya, Nick Carruthers, Peter de Boer, Wayne Drevets, John Isaac, Declan Jones, John Kemp, Hartmuth Kolb, Jeff Nye, Gayle Wittenberg, Gareth Barker, Anna Bogdanova, Heidi Byrom, Diana Cash, Annamaria Cattaneo, Daniela Enache, Tony Gee, Caitlin Hastings, Melisa Kose, Giulia Lombardo, Nicole Mariani, Anna McLaughlin, Valeria Mondelli, Maria Nettis, Naghmeh Nikkheslat, Carmine Pariante, Karen Randall, Julia Schubert, Luca Sforzini, Hannah Sheridan, Camilla Simmons, Nisha Singh, Federico Turkheimer, Vicky Van Loo, Mattia Veronese, Marta Vicente Rodriguez, Toby Wood, Courtney Worrell, Zuzanna Zajkowska, Brian Campbell, Jan Egebjerg, Hans Eriksson, Francois Gastambide, Karen Husted Adams, Ross Jeggo, Thomas Moeller, Bob Nelson, Niels Plath, Christian Thomsen, Jan Torleif Pederson, Stevin Zorn, Catherine Deith, Scott Farmer, John McClean, Andrew McPherson, Nagore Penandes, Paul Scouller, Murray Sutherland, Mary Jane Attenburrow, Jithen Benjamin, Helen Jones, Fran Mada, Akintayo Oladejo, Katy Smith, Rita Balice-Gordon, Brendon Binneman, James Duerr, Terence Fullerton, Veeru Goli, Zoe Hughes, Justin Piro, Tarek Samad, Jonathan Sporn, Liz Hoskins, Charmaine Kohn, Lauren Wilcock, Franklin Aigbirhio, Junaid Bhatti, Ed Bullmore, Sam Chamberlain, Marta Correia, Anna Crofts, Tim Fryer, Martin Graves, Alex Hatton, Manfred Kitzbichler, Mary-Ellen Lynall, Christina Maurice, Ciara O'Donnell, Linda Pointon, Peter St George Hyslop, Lorinda Turner, Petra Vertes, Barry Widmer, Guy Williams, Jonathan Cavanagh, Alison McColl, Robin Shaw, Erik Boddeke, Alison Baird, Stuart Clare, Phil Cowen, I-Shu (Dante) Huang, Sam Hurley, Simon Lovestone, Alejo Nevado-Holgado, Elena Ribe, Anviti Vyas, Laura Winchester, Madeleine Cleal, Diego Gomez-Nicola, Renzo Mancuso, Hugh Perry, Mara Cercignani, Charlotte Clarke, Alessandro Colasanti, Neil Harrison, Rosemary Murray, Jason O'Connor, Howard Mount, Federico E. Turkheimer

**Affiliations:** a Department of Neuroimaging, Institute of Psychiatry, Psychology and Neuroscience, King's College London, United Kingdom; b Department of Psychological Medicine, Institute of Psychiatry, Psychology and Neuroscience, King's College London, United Kingdom; c National Institute for Health and Research Biomedical Research Centre, South London and Maudsley NHS Foundation Trust and King’s College London, London, United Kingdom; d Department of Clinical Neurosciences, School of Clinical Medicine, University of Cambridge, Cambridge, United Kingdom; e Department of Radiology, School of Clinical Medicine, University of Cambridge, Cambridge, United Kingdom; f Department of Psychiatry, School of Clinical Medicine, University of Cambridge, Cambridge, United Kingdom; g Wolfson Brain Imaging Centre, University of Cambridge, Cambridge, United Kingdom; h Cambridgeshire and Peterborough NHS Foundation Trust, Cambridge, United Kingdom

**Keywords:** C-reactive protein, Depression, Inflammation, Microglia, PET, TSPO

## Abstract

**Background:**

Immune mechanisms have been implicated in the pathogenesis of depression. Translocator protein (TSPO)–targeted positron emission tomography (PET) has been used to assess neuroinflammation in major depressive disorder. We aimed to 1) test the hypothesis of significant case-control differences in TSPO binding in the anterior cingulate cortex, prefrontal cortex, and insula regions; and 2) explore the relationship between cerebral TSPO binding and peripheral blood C-reactive protein (CRP) concentration.

**Methods:**

A total of 51 depressed subjects with Hamilton Depression Rating Scale score >13 (median 17; interquartile range, 16–22) and 25 healthy control subjects underwent dynamic brain ^11^C-PK11195 PET and peripheral blood immune marker characterization. Depressed subjects were divided into high CRP (>3 mg/L; *n =* 20) and low CRP (<3 mg/L; *n =* 31).

**Results:**

Across the three regions, TSPO binding was significantly increased in depressed versus control subjects (η^2^_p_ = .09; *F*_1,71_ = 6.97, *p =* .01), which was not influenced by body mass index. The case-control difference was greatest in the anterior cingulate cortex (*d =* 0.49; *t*_74_ = 2.00, *p =* .03) and not significant in the prefrontal cortex or insula (*d =* 0.27 and *d =* 0.36, respectively). Following CRP stratification, significantly higher TSPO binding was observed in low-CRP depression compared with controls (*d =* 0.53; *t*_54_ = 1.96, *p =* .03). These effect sizes are comparable to prior major depressive disorder case-control TSPO PET data. No significant correlations were observed between TSPO and CRP measures.

**Conclusions:**

Consistent with previous findings, there is a modest increase in TSPO binding in depressed patients compared with healthy control subjects. The lack of a significant correlation between brain TSPO binding and blood CRP concentration or body mass index poses questions about the interactions between central and peripheral immune responses in the pathogenesis of depression.

There are a number of factors associated with major depressive disorder (MDD), and much recent research has focused on inflammation (1, 2, 3, 4, 5, 6). Increases in markers of peripheral inflammation have previously been observed in individuals with MDD compared with healthy control subjects (HCs) (6, 7, 8, 9). Furthermore, inflammation and depression often occur together—comorbidly—in the experience of patients with inflammatory diseases like rheumatoid arthritis or Crohn’s disease (10,11), during treatment with proinflammatory cytokines (12,13), and after experimental administration of a peripheral immune challenge, like typhoid vaccination (14,15). An association has also been observed between inflammation and treatment resistance to antidepressants (16,17), and adjunctive anti-inflammatory treatment has been shown to improve treatment efficacy of monoaminergic antidepressant drugs (18).

Previous studies in MDD have investigated the presence of neuroinflammation using positron emission tomography (PET) with radiotracers specific for 18-kDa translocator protein (TSPO) (1, 2, 3, 4, 5, 6). TSPO is an outer mitochondrial membrane protein, and elevations of TSPO expression have been consistently observed in microglial and macrophage populations during brain disease (19, 20, 21, 22). Despite historically being used as a marker for microglial activity, TSPO is now known to be expressed in other cell types, including reactive astrocytes and endothelial cells (23, 24, 25). These cell types have also been shown to play a role in neuroinflammatory processes (26,27). The association between TSPO expression in glial cell types involved in neuroinflammation enables the use of TSPO-specific ligand measurements to assess the presence and degree of neuroinflammation in neurological and psychiatric disease.

In studies of MDD cohorts, significantly higher TSPO binding compared with HCs has been observed in the anterior cingulate cortex (ACC) by several groups (1, 2, 3, 4), as well as higher binding in the frontal cortex (1, 2, 3, 4,6) and insula (INS) (2,4). Relationships have also been observed between TSPO binding and medication status (3), length of time treated and untreated for MDD (2), and MDD duration (2). Holmes *et al.* (1) recently found that TSPO binding was significantly greater in MDD patients with suicidal thoughts compared with those without suicidal thoughts. A significant correlation between TSPO binding and depression severity scores in MDD has also been observed (4), and a reduction in TSPO binding has been observed in MDD patients undergoing cognitive behavioral therapy (6). However, another previous analysis with a small cohort of 10 MDD subjects showed no difference in brain TSPO PET measures between MDD subjects and HCs (5). Some previous analyses did not reveal links between TSPO PET measures and clinical scores (1,5) or peripheral inflammatory markers (1,4, 5, 6).

Here, we collected clinical questionnaire data, TSPO PET brain scans, and peripheral blood immune markers from 51 depressed subjects and 25 HCs to better establish the relationship between peripheral and central inflammation in depression. ^11^C-PK11195, an isoquinoline carboxamide PET tracer specific for TSPO, was used with a dynamic PET acquisition to measure TSPO binding in the brain as a putative biomarker of central immune status. We tested the hypotheses 1) that ^11^C-PK11195 binding measured in the three aforementioned regions of interest (ROIs), namely the ACC, prefrontal cortex (PFC), and INS, is significantly increased in depression; and 2) that ^11^C-PK11195 binding is associated with blood concentration of C-reactive protein (CRP), as a biomarker of peripheral immune status, or body mass index (BMI). We also investigated whether TSPO binding is increased in treatment-resistant or unmedicated depressed subjects, and in depressed subjects with suicidal thoughts compared with those without, in an attempt to replicate previous results.

## Methods and Materials

### Participants

A total of 51 depressed subjects (36 women/15 men; mean age: 36.2 ± 7.4 years) and 25 age-matched HCs (14 women/11 men; mean age: 37.3 ± 7.8 years) were recruited from a network of clinical research sites in the United Kingdom as part of the BIODEP (Biomarkers in Depression) study (NIMA consortium; https://www.neuroimmunology.org.uk/biodep/). Depressed individuals 25 to 50 years of age (inclusive) with a total Hamilton Depression Rating Scale (HDRS) (28) score >13 were included. Depressed subjects were recruited and stratified into low/high CRP groups using a blood CRP concentration threshold of 3 mg/L, resulting in 31 low-CRP subjects and 20 high-CRP subjects. During post hoc analysis, depressed subjects not on treatment for at least 6 weeks prior to screening with HDRS score >17 were classified as untreated, resulting in 9 untreated subjects. Depressed subjects who were still moderately depressed with HDRS score >13 despite more than 6 weeks treatment with one or more monoaminergic antidepressant were classified as treatment resistant, resulting in 33 treatment-resistant subjects. Nine subjects were not included in the treatment status groupings because their treatment status or response was unknown. Depressed subjects with a score of 2 or higher on the suicide item of the HDRS were classified as having suicidal thoughts, and those with a score of 0 on the suicide item were classified as having no suicidal thoughts, resulting in 12 subjects with and 20 subjects without suicidal thoughts.

All depressed subjects and HCs passed the following exclusion criteria: a lifetime history of other neurological disorders, active drug and/or alcohol abuse, participation in clinical drug trials within the previous year, concurrent medication or medical disorder that could compromise the interpretation of results, and pregnancy or breastfeeding. HCs had no personal history of clinical depression requiring treatment and were mean age–matched with the depressed group. Additional information about subject comorbidities can be found in the Supplement. The study was approved by the National Research Ethics Service Committee East of England – Cambridge Central (REC reference:15/EE/0092) and the UK Administration of Radioactive Substances Advisory Committee. All subjects gave written informed consent prior to data collection.

### Clinical Assessments

All subjects underwent an in-depth clinical evaluation that included the following psychiatric assessments: the HDRS; Structured Clinical Interview for DSM-5, Research Version; Beck Depression Inventory; Spielberger State-Trait Anxiety Inventory; Chalder Fatigue Scale; Snaith–Hamilton Pleasure Scale; and Perceived Stress Scale. Medical and family history was collected by a trained member of the research team. A venous blood sample was collected for assessing CRP level, as previously published (29). Venous blood was sampled from an antecubital vein between 08:00 am and 10:00 am on the day of clinical assessment. Participants had fasted for 8 hours, had refrained from exercise for 72 hours, and had been lying supine for 0.5 hour prior to venipuncture. Blood was collected into a serum-separating tube, completely and gently inverted 10 times, allowed to coagulate for a minimum of 30 minutes and maximum of 60 minutes, and centrifuged at 1600 relative centrifugal force for 15 minutes. A total of 1 mL of serum was then transferred with a pipette to a serum tube and sent on the day of collection to a central lab for high-sensitivity CRP assay via turbidimetric detection using a Beckman Coulter AU analyzer (Beckman Coulter, Brea, CA), with rabbit anti-CRP-antibodies coated on latex particles.

### TSPO PET Data Acquisition and Analysis

Dynamic PET data acquisition was performed on a GE SIGNA PET/MR (GE Healthcare, Waukesha, WI) for 60 minutes after ^11^C-PK11195 injection (mean = 361 ± 53 MBq). Attenuation correction included the use of a multisubject atlas method (30,31) and improvements for the magnetic resonance imaging (MRI) brain coil component (32). Other data corrections (dead time, randoms, normalization, scatter, sensitivity, and decay) were as implemented on the scanner. Dynamic sinograms were reconstructed into 128 × 128 × 89 arrays (2.0 × 2.0 × 2.8 mm voxel size) using time-of-flight ordered subsets expectation maximization, with 6 iterations, 16 subsets, and no smoothing. Examples of TSPO PET images from 10 to 60 minutes for 1 HC and 1 depressed subject are shown in Figure S1. During PET data acquisition, all subjects also had a volumetric, high-resolution T1-weighted brain MRI (BRAVO), which was used for PET data processing.

Brain extraction, tissue segmentation, alignment of the MRI and PET data, and motion correction were performed using MIAKAT (version 4.2.6; http://www.miakat.org/MIAKAT2/index.html) software. MIAKAT is implemented in MATLAB (version R2015b; The MathWorks, Inc., Natick, MA) and uses tools from SPM12 and FSL (version 5.0.9). Data quality control was performed through visual inspection of the outputs of the MRI and PET processing steps. Experimental variables including injected activity, total motion during PET, and maximum interframe motion during PET were recorded for all subjects (Table 1). The CIC v2.0 neuroanatomical atlas (33) was coregistered to the image space of each subject and used to extract time-activity curves from a subset of 24 ROIs based on their relevance to MDD. A simplified reference tissue model using a supervised clustering reference region approach (34) was used to quantify ^11^C-PK11195 binding as relative binding potential (BP_ND_) in three primary bilateral ROIs (ACC, PFC, and INS), which are shown in Figure S1C. Global increases in TSPO could induce an underestimation of BP_ND_ measures in the target regions when using a reference tissue model. Blood input functions still provide accurate quantification as long as changes in tracer extraction or free plasma fractions (35) or perfusion (36) are taken into account. Reference region approaches, once carefully validated for each tracer, can provide reliable parameters while being robust to changes in plasma radioactivity and the changes in immune status that characterize this cohort [see (37) for an example]. Example time-activity curves for ACC of 1 HC and 1 depressed subject are shown in Figures S2A and S2B, respectively. The supervised approach uses a predetermined set of kinetic tissue classes to identify voxels with kinetic behavior closest to that of healthy gray matter. The time course of the activity averaged over the identified voxels, weighted by the gray matter kinetic class scaling coefficient per voxel, is used as the reference input function. Use of a supervised cluster analysis technique for extracting reference tissue has previously been cross-validated and shown to be a reliable method for quantifying brain ^11^C-PK11195 (38). The three ROIs were selected based on previous findings (1,4), which were originally motivated by their role in mood regulation (39,40), together with previous suggestions of the involvement of the ACC in the link between inflammation and depression (14,41, 42, 43, 44, 45). The PFC region used in this analysis is the aggregate of the medial and dorsolateral frontal cortex regions, and the ACC region is the aggregate of the ventral cingulate subcallosal gyrus, anterior cingulate gyrus, and dorsal anterior cingulate regions from the CIC v2.0 neuroanatomical atlas.

**Table 1:** Demographic and Clinical Characteristics for Depressed and Healthy Control Subjects

Variable	Depressed Subjects (*n* = 51)	Healthy Control Subjects (*n* = 25)	*p* Value
Age, Years, Mean (SD)	36.2 (7.3)	37.3 (7.8)	.561
Male, *n* (%)	15 (29%)	11 (44%)	.208
Weight, kg, Mean (SD)	80.3 (14.4)	73.7 (15.1)	.102
BMI, kg/m^2^, Mean (SD)			
All	27.2 (4.0)	24.2 (4.8)	.001^a^
High CRP	30.0 (3.8)	NA	NA
Low CRP	26.2 (3.4)	NA	NA
CRP, mg/L, *n*, Mean (SD)			
All	51, 2.9 (2.8)	25, 1.1 (0.9)	<.001^a^
High CRP	20, 5.6 (2.6)	1, 3.9 (NA)	NA
Low CRP	31, 1.1 (0.7)	24, 1.0 (0.8)	NA
Medication Status, *n* (%)			
Untreated	9 (17.5%)	NA	NA
Treatment resistant	33 (65%)	NA	NA
No grouping	9 (17.5%)	NA	NA
HDRS, Mean (SD)	18.5 (3.7)	0.6 (0.9)	<.001^a^
Dose of Radioactive Isotope Injected, MBq, Mean (SD)	360.3 (53.2)	376.2 (44.8)	.138
Injected Mass, μg, Mean (SD)	3.0 (1.6)	3.4 (1.8)	.486
Specific Activity, GBq/μmol, Mean (SD)	50.80 (21.33)	50.56 (25.94)	.711
Total Motion, mm, Mean (SD)	7.76 (3.75)	7.27 (3.39)	.562
Max Interframe Motion, mm, Mean (SD)	1.62 (0.90)	1.45 (0.68)	.532
Scan Start Time, hh:mm:ss	15:14:44	14:58:05	.761

BMI, body mass index; CRP, C-reactive protein; HDRS, Hamilton Depression Rating Scale; NA, not applicable.

a *p* < .05.

### Statistical Analysis

SPSS (version 24.0; IBM, Armonk, NY) was used to perform all statistical analyses. Normality of the data was tested using Shapiro-Wilk’s *W* test. The difference in BP_ND_ between depressed and HC groups across the ACC, PFC, and INS regions was investigated using analysis of variance by performing a repeated-measures general linear model analysis to test for case-control differences, while covarying for age, sex, and BMI. Current and past tobacco use was also investigated as a covariate. Group differences in experimental variables (subject age, body weight, injected activity, total motion, maximum interframe motion) and BP_ND_ in individual ROIs were further investigated using the independent-samples Mann-Whitney *U* test and 1-tailed independent-samples *t* test, respectively. The relationship between sex and depression diagnosis was assessed using a χ^2^ test. Group differences were evaluated with 5% probability of type I error (α = .05). The relationships between CRP concentration and BP_ND_, and between psychiatric assessment scores and BP_ND_ in the three primary ROIs, were assessed using Spearman’s correlation. Estimated sample sizes required to achieve significant group differences in the PFC and INS regions were calculated given the observed effect sizes, with α = .10 and β = .20. Nominal *p* values are reported without correction for multiple comparisons. The ACC, PFC, and INS regions were selected based on previous positive findings, so a global null hypothesis and ensuing multiple correction is not appropriate (46).

## Results

### Demographic and Clinical Characteristics

Demographic, clinical, and PET scan details for HCs and depressed subjects are given in Table 1. There are significant differences in BMI (*p =* .001), serum CRP concentration (*p <* .001), and HDRS (*p <* .001) between depressed subjects and HCs. No other significant differences between HCs and depressed subjects were observed for PET imaging parameters.

### TSPO Tracer Binding Comparisons Between HCs and Subjects With Depression

**Figure 1:**
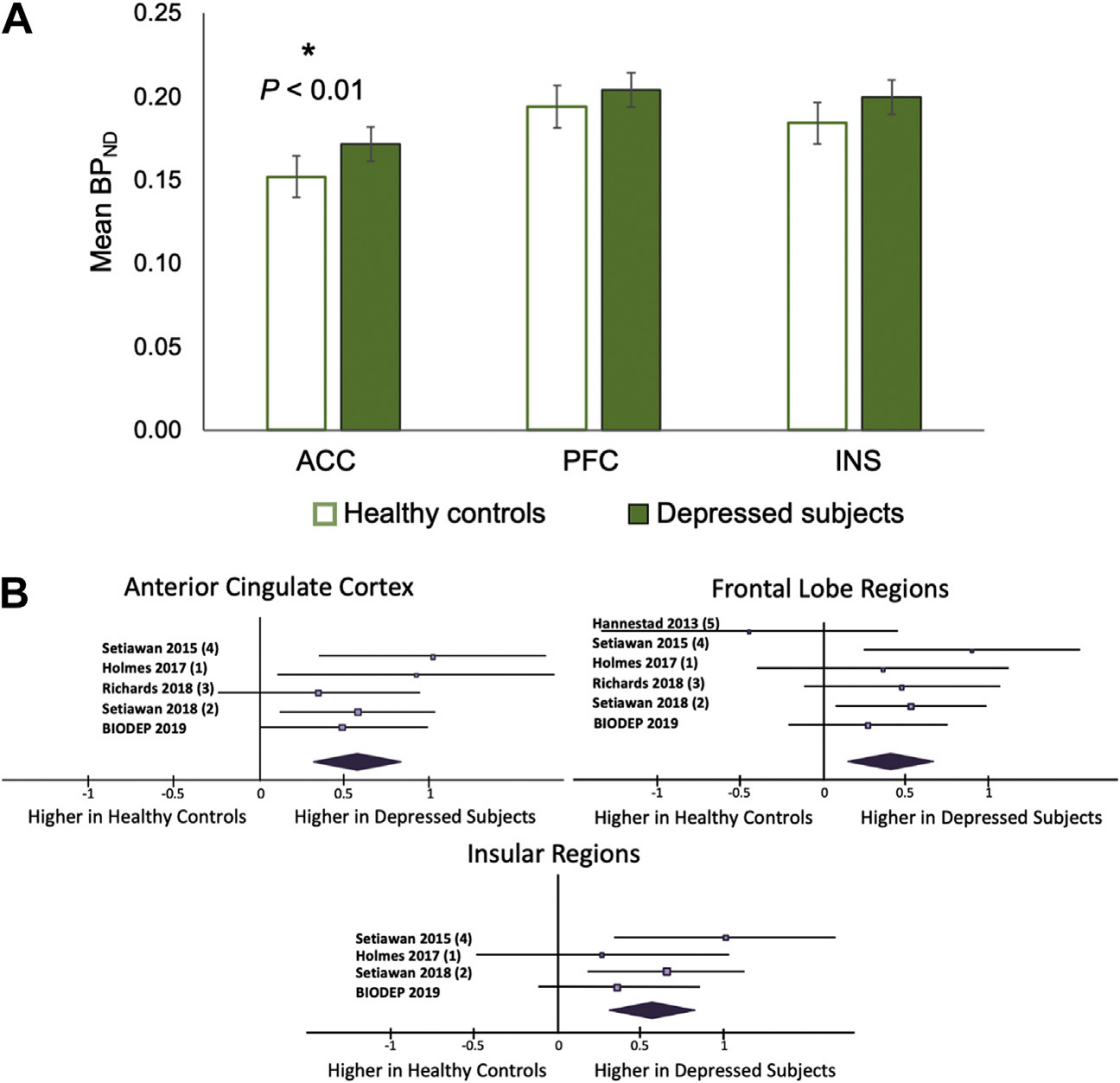
**(A)** Case-control differences in mean ^11^C-PK11195 binding potential measurements in anterior cingulate cortex (ACC), prefrontal cortex (PFC), and insula (INS) regions between healthy control and depressed subjects. Error bars represent SE. The analyses are corrected for age, sex, and body mass index. Significant differences (*p <* .05) are indicated by an asterisk. **(B)** Forest plot summarizing the current results in the context of previous translocator protein positron emission tomography results from case-control studies of depression in the ACC, frontal lobe regions, and INS regions. BP_ND_, relative binding potential.

The analysis of variance revealed a significant case-control difference in BP_ND_ across the three primary ROIs (η^2^_p_ = .09; *F*_1,71_ = 6.97, *p =* .01) (Figure 1A). Increased BP_ND_ across the three primary ROIs in depressed subjects compared with HCs remained significant with the addition of tobacco use as a covariate (*F*_1,69_ = 5.57, *p =* .02), which was not found to be a significant contributor to the statistical model (*F*_1,69_ = 0.34, *p =* .56). A significantly higher average BP_ND_ was observed in the ACC in depressed subjects (mean = 0.17, SD = 0.04) compared with HCs (mean = 0.15, SD = 0.05 [*d =* 0.49; *t*_74_ = 2.00, *p =* .03]), which remained significant when covaried for age, sex, and BMI (η^2^_p_ = .11; *F*_1,71_ = 9.07, *p =* .004). There were no other significant differences in BP_ND_ between groups in the PFC (*d =* 0.27; *t*_74_ = 1.11, *p =* .13) or INS (*d =* 0.36; *t*_74_ = 1.48, *p =* .07) regions, at nominal 5% probability of type I error. These results are shown in the context of previously reported case-control differences in ^11^C-PK11195 binding in a forest plot in Figure 1B. Estimated sample sizes required to achieve significant differences with the observed effect sizes are *n =* 252 for the PFC and *n =* 116 for the INS, assuming an equal number of subjects in the case and control groups.

### Relationships Between TSPO Tracer Binding and CRP

**Figure 2:**
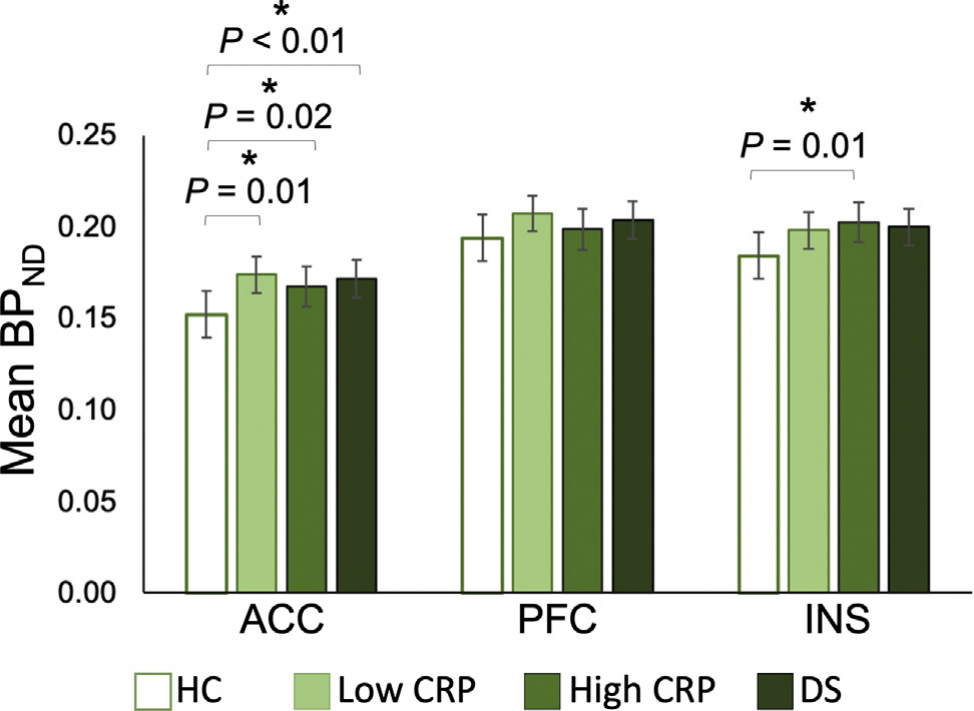
Case-control differences in mean ^11^C-PK11195 binding potential measurements in the anterior cingulate cortex (ACC), prefrontal cortex (PFC), and insula (INS) regions between healthy control subjects (HC), low C-reactive protein (CRP) depression, high-CRP depression, and all depressed subjects (DS). Error bars represent SE. Analyses are corrected for age, sex, and body mass index. Significant differences (*p <* .05) are indicated by an asterisk. BP_ND_, relative binding potential.

**Figure 3:**
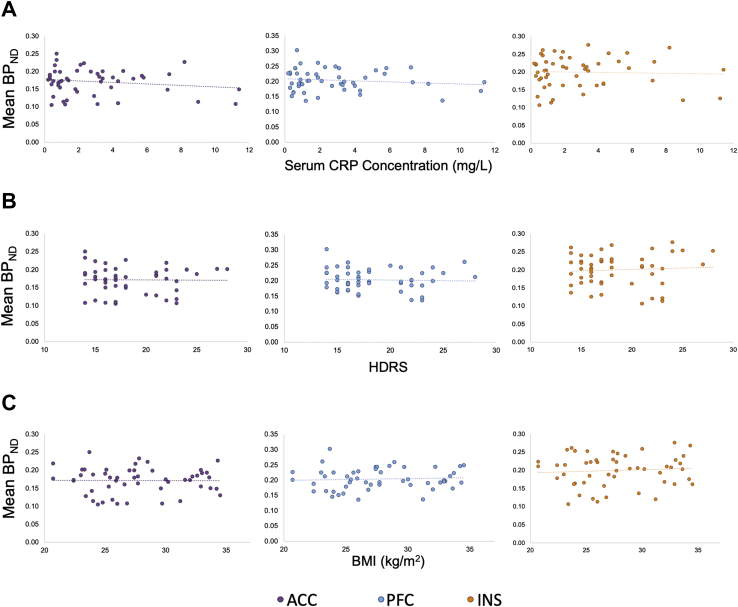
Scatterplots of ^11^C-PK11195 binding potential measurements (y-axis) for the anterior cingulate cortex (ACC), prefrontal cortex (PFC), and insula (INS) regions vs. **(A)** serum C-reactive protein (CRP) concentration (mg/L), **(B)** Hamilton Depression Rating Scale (HDRS) and **(C)** body mass index (BMI, kg/m^2^) in depressed subjects.

A significantly higher average BP_ND_ was observed in the ACC in low-CRP depressed subjects compared with HCs (*d =* 0.53; *t*_54_ = 1.96, *p =* .03) before correcting for age, sex, and BMI. Significantly higher average BP_ND_ was observed in the ACC in high-CRP depressed subjects compared with HCs (η^2^_p_ = .13; *F*_1,40_ = 5.74, *p =* .02), in the ACC in low-CRP depressed patients compared with HCs (η^2^_p_ = .15; *F*_1,51_ = 8.82, *p =* .01), and in the INS in high-CRP depressed subjects compared with HCs (η^2^_p_ = .14; *F*_1,40_ = 6.68, *p =* .01) after correcting for age, sex, and BMI (Figure 2). No other significant differences were observed when the depressed group was stratified by CRP concentration. No significant correlations were observed between the ACC, PFC, or INS BP_ND_ and the CRP (Table 2), HDRS, or BMI (Figure 3).

**Table 2:** Correlation Between Regional TSPO Binding Potential and CRP in Depressed Patients and Healthy Controls

Region	CRP	
	Depressed Subjects	Healthy Control Subjects
ACC	*r* = −.047 *p =* .745	*r* = −.162 *p =* .438
PFC	*r* = −.073 *p =* .610	*r* = .271 *p =* .190
INS	*r* = .053 *p =* .710	*r* = .243 *p =* .241

ACC, anterior cingulate cortex; CRP, C-reactive protein; INS, insula; PFC, prefrontal cortex; TSPO, translocator protein.

### Post Hoc Investigations of TSPO Tracer Binding and Clinical Variables

No significant correlations were observed between BP_ND_ and HDRS scores, as the primary score to investigate disease severity in depression (Figure 3B). Further exploratory analysis showed no significant correlations between BP_ND_ and other psychiatric assessment scores including the Beck Depression Inventory, Spielberger State-Trait Anxiety Rating Scale, Chalder Fatigue Scale, Snaith–Hamilton Pleasure Scale, and Perceived Stress Scale. Differently from previous studies, no significant differences were observed between HCs and patients with treatment-resistant depression, between HCs and subjects with untreated depression (Figure S3A), between HCs and depressed subjects with suicidal thoughts, or between depressed subjects with and without suicidal thoughts (Figure S3B).

A complete report of the primary statistical analysis results is included in Tables S1 to S3. Correlation matrices showing the relationship between regional BP_ND_, CRP, HDRS, and BMI in depressed subjects and HCs are included in Tables S4 and S5, respectively.

## Discussion

This study successfully replicates previous results of increased TSPO binding in depressed subjects, irrespective of depression severity or medication status, compared with HCs in one of the largest-to-date samples of TSPO PET data collected together with peripheral inflammatory markers in depression (47). Consistent with the study hypothesis, we observed an increase in TSPO binding in depressed subjects compared with HCs across the three primary ROIs (ACC, PFC, and INS). However, we did not observe any significant correlation between brain TSPO PET measures and serum CRP concentration, BMI, or clinical scores. We did not replicate previous findings of increased TSPO binding in untreated subjects compared with HCs (3) or in subjects with suicidal thoughts compared with subjects without suicidal ideation (1). We observed greater variability in BP_ND_ values in all three primary ROIs in the HCs compared with depressed subjects.

TSPO PET studies may be difficult to compare owing to use of different radiotracers and quantification measures (35). Even so, recent studies (1, 2, 3, 4) along with ours indicate that there is increased TSPO binding in depressed subjects compared with HCs, in which the meta-analytic effect size is medium on average over all studies (overall Hedges’ *g* for the ACC = 0.60, for the PFC = 0.41, for the INS = 0.57). Generally, these results provide robust support for the presence of somewhat greater central nervous system inflammation in depressed subjects compared with HCs. Our result of no association between CRP and TSPO binding is also consistent with recent literature (1,3,4,6) and suggests that, although an association has been observed between increased peripheral inflammation and MDD (48), CRP as a marker for peripheral inflammation is not associated with central inflammation putatively measured with TSPO PET. BMI is one of the main contributing factors to chronic low-grade inflammation (49) and has been identified as a confounding factor in TSPO PET studies (50). However, depression is associated with peripheral inflammation even after adjusting for BMI (29,51,52). The lack of association between BMI and PET signal further confirms that peripherally produced inflammation does not translate directly into central inflammation. Consistent findings of weak central inflammation in depressed subjects compared with HCs, and the lack of a direct association between measures of central and peripheral inflammation, do not seem to match the associations between peripheral and central inflammation observed in animal models (53). Previous findings have posed the model of depression involving direct induction of central immune activation by peripheral cytokines, which in turn could lead to a biochemical cascade that eventually leads to depressive symptoms (54,55). However, if this model were to apply in our cohort, we would expect less variability in the TSPO result of the depressed group and would expect to observe an association between central (TSPO PET) and peripheral (CRP) inflammatory measures, which we do not. In fact, most animal models of inflammation-induced sickness behavior are characterized by a leaky blood-brain barrier (53), and MDD has only been observed to exhibit a leaky blood-brain barrier in a few cases (56), suggesting that these animal models may be inappropriate for representing the general relationship between the central nervous system and the periphery in MDD. TSPO PET results investigating the link between central and peripheral inflammation by peripheral immune challenge in humans are also contradictory. One study showed that injection of *Escherichia coli* lipopolysaccharide significantly increased measures of brain TSPO (57). Another showed that immune challenge with interferon alpha exhibited no change in brain TSPO (58), and both studies showed no link between peripheral inflammation, measured by serum CRP, and brain TSPO (57,58).

This does not imply that peripheral inflammation and depression are otherwise unrelated. Increased peripheral (51,59) and central (1,3,4,6) inflammatory markers have been separately observed in MDD subjects that do not present with inflammatory comorbidities, which suggests that both peripheral and central inflammation may play a part in the development or progression of MDD. However, the mechanism linking peripheral cytokine activity and central immunity is still unclear; our results, along with those from previous groups, suggest that this complex relationship warrants further investigation in more realistic settings. Interestingly, some of our findings became more significant or apparent when controlling the statistical analyses for BMI. It is well acknowledged that the metabolic and immune systems are strongly integrated and that higher BMI is associated with higher levels of peripheral inflammation, including higher CRP levels (60). The finding of significantly higher TSPO binding in depressed subjects even after correcting for BMI again suggests that a peripheral metabolic–immune dysfunction per se is not sufficient to induce increase in TSPO expression or development of depression, and other factors, such as blood-brain barrier permeability, may be relevant for a better understanding of the link between peripheral metabolic–immune dysfunction and depression.

### Limitations

This study has some limitations. The greater variability of the BP_ND_ values for all regions in the HC group compared with depressed group presented here is surprising. A possible explanation for this is the higher proportion of males in the HC group, who have previously shown to have higher variance in BP_ND_. Recruitment of a truly healthy control group is challenging, and questionnaires about current and past medical problems were analyzed for all subjects to attempt to exclude depressed subjects and HCs with potentially confounding medical comorbidities. However, some subjects had a history of medical conditions that could be associated with increased inflammation, such as recovery from influenza within the month before data collection or ongoing mild asthma. Full details about subject comorbidities are included in the Supplement. No drug testing was performed prior to data collection, which could have resulted in inclusion of subjects exhibiting confounding drug effects. The TSPO PET signal may be difficult to interpret at the cellular level because TSPO is not specific to microglia. TSPO is also upregulated in other cell types during disease (22,24,61). Other cell types that express TSPO include macrophages, astrocytes, epithelial cells, and vascular endothelial cells, and they all have a role in central nervous system immunity (61,62). More recent evidence has also shown that microglial expansion does correspond to TSPO binding in tissue during disease (24). The future availability of more specific markers for microglial activation and neuroinflammation (63, 64, 65, 66, 67, 68, 69, 70), to be used in place of or in parallel to TSPO ligands, will substantially help the interpretation of future results in the area of neuroinflammation and depression. ^11^C-PK11195 has a lower sensitivity to TSPO compared with second-generation TSPO tracers, which could explain why we did not replicate previous findings of increased TSPO binding in untreated depressed subjects compared with controls (3). However, the primary result of greater TSPO binding in depressed compared with healthy subjects does not appear to be tracer specific, as previous significant results using ^11^C-PK11195 in MDD have also been reported (1). Depressed subjects included here are moderately depressed, as characterized by an HDRS score >13, although not all fulfill the diagnostic criteria for MDD or for an ongoing major depressive episode, which could also contribute to differing results compared with findings from previous studies.

### Conclusions

We have contributed to the growing body of work (1, 2, 3, 4,6) that continues to support a relationship between neuroinflammation and depression. However, the effect size of case-control differences in the TSPO PET signal is small and not correlated with peripheral CRP concentrations, suggesting the scope for future, more mechanistic studies that will require novel PET radioligands with specific brain immune targets.
