# Supplementary material for: A Modest Increase in 11C-PK11195-Positron Emission Tomography TSPO Binding in Depression Is Not Associated With Serum C-Reactive Protein or Body Mass Index
Source: Biol Psychiatry Cogn Neurosci Neuroimaging. 2021 Jul;6(7):716–24. doi: 10.1016/j.bpsc.2020.12.017 (PMC8264953; doi:10.1016/j.bpsc.2020.12.017)
Supplement: Supplementary Materials [file mmc1.pdf]

A Modest Increase in ^{11}C-PK11195 Positron Emission Tomography TSPO Binding in Depression Is Not Associated With Serum C-Reactive Protein or Body Mass Index

Supplemental Information

Supplemental Methods

Information on subject comorbidities

One control subject had visual disturbances and a small heart murmur. Three other controls had mild respiratory disorders, four had gastrointestinal issues, six had mild musculoskeletal issues/pain, three had history of renal issues, one had mild eczema, three had history of thyroid issues, one had a history of anaemia during pregnancy, and four had recently (within the previous month) recovered from influenza. One depressed subject had pars planitis, one had history of minor heartbeat inconsistency, one had hypertension, thirteen had history of or ongoing mild asthma, ten had gastrointestinal issues, one had previously recovered from shingles, nineteen had musculoskeletal issues/pain, nine had a history of renal issues, eight had mild dermatological issues, six had a history of thyroid issues, three had history of anaemia, one had a condition that can cause abnormal blood clotting, five had recently (within the previous month) recovered from influenza, one recently recovered from glandular fever, one from giardia, one from bronchitis, and one from tonsilitis. Three subjects reported having flu-like symptoms within the previous three months and one reported having slower recovery than usual from the flu.

Supplemental Figures

Figure S1. 11C-PK11195 PET images from 10 to 60 minutes for one healthy control (A) and one depressed subject (B). C:Anterior cingulate cortex (ACC), prefrontal cortex (PFC), and insula (INS) regions overlaid on MNI152 brain template.

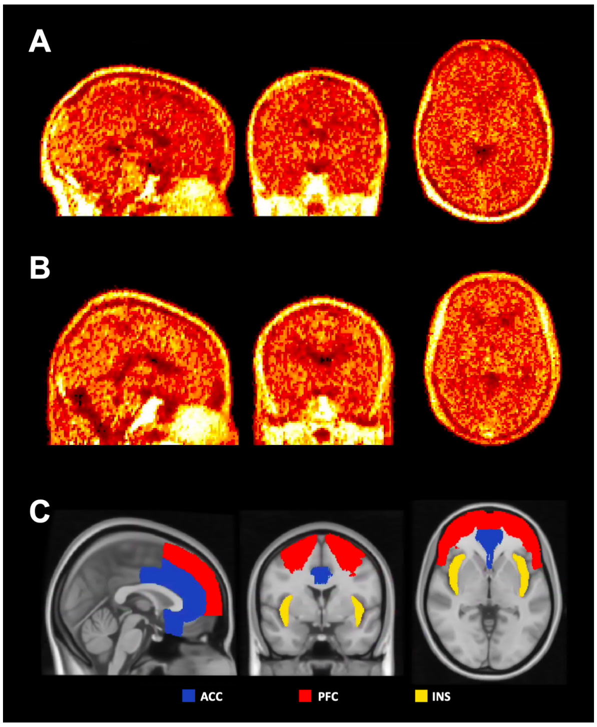

Figure S2. Example time activity curve for ACC of a healthy control (A) and depressed subject (B).

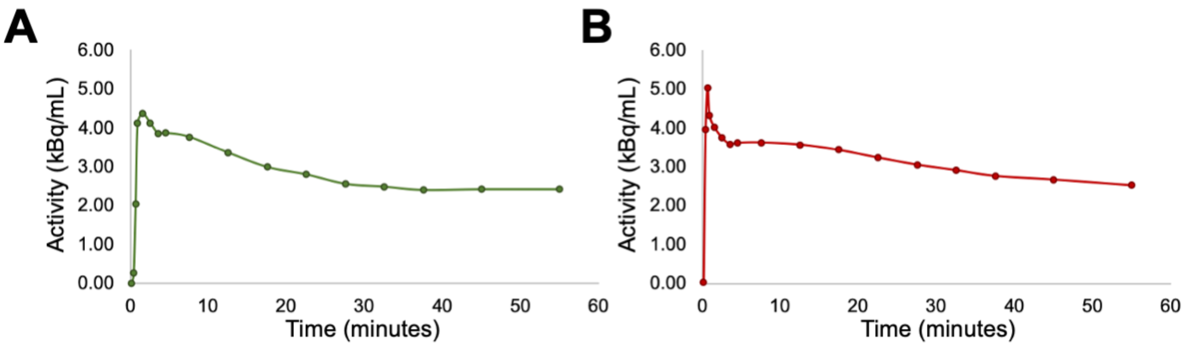

Figure S3. A. Group differences in mean ^{11}C-PK11195 binding potential measurements in anterior cingulate cortex (ACC), prefrontal cortex (PFC), and insula (INS) regions between healthy controls (HC), treatment resistant depression, and untreated depression. **B.** Group differences in mean ^{11}C-PK11195 binding potential measurements in ACC, PFC, and INS regions between healthy controls, depressed subjects without suicidal thoughts, and depressed subjects with suicidal thoughts. Error bars represent standard error.

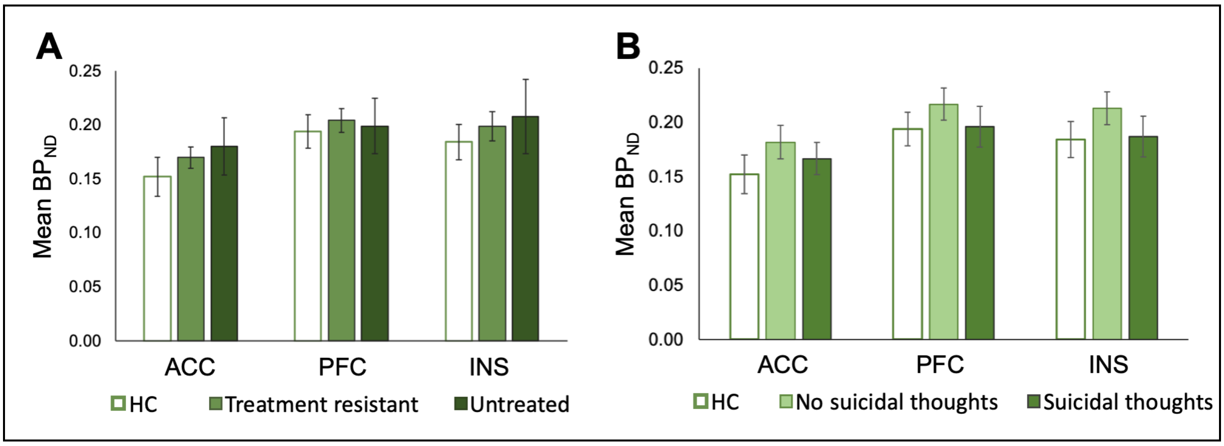

Supplemental Tables

Table S1. One-tailed t-statistic results comparing regional TSPO binding potentials (BP_{ND}) in anterior cingulate cortex (ACC), prefrontal cortex (PFC), and insula (INS) brain regions between groupings of depressed subjects (DS) (all DS, high C-reactive protein (CRP), low CRP) and healthy controls (HC).

Region		t-statistic	P-Value	Mean±SD	
				DS	HC
ACC	All DS	t(74)=1.996	0.025	1.171±0.036	1.152±0.046
	High CRP	t(43)=1.236	0.112	1.168±0.035	
	Low CRP	t(54)=1.958	0.028	1.174±0.037	
PFC	All DS	t(74)=1.114	0.134	1.204±0.035	1.194±0.040
	High CRP	t(43)=0.435	0.333	1.199±0.030	
	Low CRP	t(54)=1.284	0.102	1.207±0.038	
INS	All DS	t(74)=1.484	0.071	1.200±0.044	1.184±0.042
	High CRP	t(43)=1.396	0.085	1.202±0.045	
	Low CRP	t(54)=1.215	0.115	1.198±0.043	

Table S2. Analysis of variance (ANOVA) results comparing regional TSPO binding potentials (BP_{ND}) in anterior cingulate cortex (ACC), prefrontal cortex (PFC), and insula (INS) brain regions between groupings of depressed subjects (DS) (all DS, high C-reactive protein (CRP), low CRP) and healthy controls (HC).

Region		F-statistic	P-Value
**Controlled for age**			
All regions	All DS	F(1,73)=3.421	0.068
	High CRP	F(1,42)=1.413	0.241
	Low CRP	F(1,53)=3.024	0.088
ACC	All DS	F(1,73)=4.880	0.030
	High CRP	F(1,42)=1.559	0.219
	Low CRP	F(1.53)=4.892	0.031
PFC	All DS	F(1,73)=1.156	0.286
	High CRP	F(1,42)=0.191	0.665
	Low CRP	F(1,53)=1.364	0.248
INS	All DS	F(1,73)=2.567	0.113
	High CRP	F(1,42)=1.929	0.172
	Low CRP	F(1,53)=1.769	0.189
**Controlled for age and sex**			
All regions	All DS	F(1,72)=5.590	0.021
	High CRP	F(1,41)=3.752	0.060
	Low CRP	F(1,52)=4.102	0.048
ACC	All DS	F(1,72)=6.599	0.012
	High CRP	F(1,41)=3.376	0.073
	Low CRP	F(1,52)=5.720	0.020
PFC	All DS	F(1,72)=2.589	0.112
	High CRP	F(1,41)=0.999	0.323
	Low CRP	F(1,52)=2.261	0.139
INS	All DS	F(1,72)=3.973	0.050
	High CRP	F(1,41)=4.551	0.039
	Low CRP	F(1,52)=2.365	0.130
**Controlled for age, sex, and BMI**			
All regions	All DS	F(1,71)=6.971	0.010
	High CRP	F(1,40)=7.224	0.010
	Low CRP	F(1,51)=5.708	0.021
ACC	All DS	F(1,71)=9.067	0.004
	High CRP	F(1,40)=5.741	0.021
	Low CRP	F(1,51)=8.815	0.005
PFC	All DS	F(1,71)=3.858	0.053
	High CRP	F(1,40)=3.707	0.061
	Low CRP	F(1,51)=3.460	0.069
INS	All DS	F(1,71)=3.848	0.054
	High CRP	F(1,40)=6.678	0.014
	Low CRP	F(1,51)=2.628	0.111

Table S3. Correlation between regional TSPO binding potentials and experimental variables in anterior cingulate cortex (ACC), prefrontal cortex (PFC), and insula (INS) regions in depressed subjects (DS) and healthy controls (HC)

Experimental Variable	Region, r Value (P Value)*			
**Variable only**		**ACC**	**PFC**	**INS**
CRP	All DS	-0.047(0.745)	-0.073(0.610)	0.053(0.710)
	High CRP	-0.127(0.593)	-0.102(0.670)	-0.050(0.833)
	Low CRP	0.034(0.856)	0.064(0.734)	0.031(0.867)
	All HC	0.162(0.438)	0.271(0.190)	0.243(0.241)
HDRS	All DS	-0.009(0.951)	-0.035(0.812)	0.080(0.582)
	High CRP	0.486(0.035)*	0.223(0.359)	0.501(0.029)*
	Low CRP	-0.247(0.180)	-0.088(0.638)	-0.186(0.317)
**Controlled for age**				
CRP	All DS	-0.171(0.241)	-0.184(0.207)	-0.035(0.812)
	High CRP	-0.276(0.252)	-0.173(0.478)	-0.218(0.371)
	Low CRP	-0.024(0.901)	0.098(0.605)	0.044(0.816)
	All HC	0.189(0.377)	0.170(0.426)	0.275(0.194)
HDRS	All DS	-0.052(0.723)	-0.047(0.746)	0.041(0.781)
	High CRP	0.294(0.236)	0.112(0.658)	0.434(0.072)
	Low CRP	-0.233(0.216)	-0.110(0.564)	-0.219(0.245)
**Controlled for age and sex**				
CRP	All DS	-0.142(0.334)	-0.127(0.391)	-0.002(0.988)
	High CRP	-0.193(0.458)	-0.194(0.456)	-0.124(0.634)
	Low CRP	0.022(0.911)	0.098(0.611)	0.043(0.827)
	All HC	0.143(0.515)	0.123(0.577)	0.229(0.294)
HDRS	All DS	-0.077(0.605)	-0.103(0.487)	0.018(0.901)
	High CRP	0.279(0.278)	0.086(0.743)	0.427(0.087)
	Low CRP	-0.258(0.177)	-0.195(0.310)	-0.247(0.197)
**Controlled for age, sex, and BMI**				
CRP	All DS	-0.155(0.298)	-0.125(0.403)	-0.042(0.778)
	High CRP	-0.213(0.428)	-0.193(0.475)	-0.124(0.646)
	Low CRP	0.064(0.747)	0.072(0.714)	0.012(0.950)
	All HC	0.267(0.229)	0.243(0.275)	0.316(0.152)
HDRS	All DS	-0.077(0.607)	-0.104(0.486)	0.022(0.884)
	High CRP	0.354(0.178)	0.073(0.788)	0.432(0.095)
	Low CRP	-0.269(0.167)	-0.190(0.333)	-0.242(0.215)

Table S4. Correlation between regional TSPO binding potential (ACC: anterior cingulate cortex; PFC: prefrontal cortex; INS: insula) and C-reactive protein (CRP), Hamilton depression rating scale (HDRS), and body mass index (BMI) in depressed subjects.

	**PFC**	**INS**	**CRP**	**HDRS**	**BMI**
**ACC**	r=0.483<br>*P*<0.001	r=0.757<br>*P*<0.001	r=-0.047<br>*P*=0.745	r=-0.009<br>*P*=0.951	r=-0.19<br>*P*=0.895
**PFC**		r=0.456<br>*P*=0.001	r=-0.073<br>*P*=0.610	r=-0.035<br>*P*=0.812	r=0.112<br>*P*=0.434
**INS**			r=0.053<br>*P*=0.710	r=0.080<br>*P*=0.582	r=0.044<br>*P*=0.760
**CRP**				r=0.302<br>*P*=0.033	r=0.462<br>*P*=0.001
**HDRS**					r=0.012<br>*P*=0.935

Table S5. Correlation between regional TSPO binding potential (ACC: anterior cingulate cortex; PFC: prefrontal cortex; INS: insula) and C-reactive protein (CRP), Hamilton depression rating scale (HDRS), and body mass index (BMI) in healthy controls.

	**PFC**	**INS**	**CRP**	**HDRS**	**BMI**
**ACC**	r=0.755<br>*P*<0.001	r=0.796<br>*P*<0.001	r=-0.162<br>*P*=0.438	r=0.204<br>*P*=0.327	r=-0.045<br>*P*=0.831
**PFC**		r=0.548<br>*P*=0.005	r=0.271<br>*P*=0.190	r=0.001<br>*P*=0.995	r=0.003<br>*P*=0.990
**INS**			r=0.243<br>*P*=0.241	r=0.401<br>*P*=0.047	r=0.059<br>*P*=0.780
**CRP**				r=0.322<br>*P*=0.117	r=0.323<br>*P*=0.116
**HDRS**					r=-0.026<br>*P*=0.903
